# Oral ATP treatment in alternating hemiplegia of childhood: a case report and review

**DOI:** 10.3389/fmed.2024.1433217

**Published:** 2025-01-07

**Authors:** Marco Carrozzi, Maria Elisa Morelli, Mario Cirino, Alessandra Maestro, Gilda Paternuosto, Giulia Benericetti, Giada Bennati, Maura Bin, Anna Flamigni, Federico Pigato, Natalia Maximova, Egidio Barbi, Davide Zanon

**Affiliations:** ^1^Institute for Maternal and Child Health Burlo Garofolo (IRCCS), Trieste, Italy; ^2^Department of Medicine, Surgery and Health Sciences, University of Trieste, Trieste, Italy

**Keywords:** adenosine-5′-triphosphate, ATP, alternating hemiplegia of childhood, rare disease, compound, clinical galenic

## Abstract

Alternating hemiplegia of childhood (AHC) is a rare neurological disorder that usually manifests before 18 months of age and is characterized by recurrent, alternating episodes of hemiparesis with variable frequency and can last from a few minutes to several days. We present a case of AHC in a little girl carrying a sporadic mutation in the ATP1A3 gene (p.Glu815Lys) refractory to flunarizine and non-compliant to topiramate due to adverse effects treated with oral compound of adenosine-5′-triphosphate (ATP) capsules. Outcome was evaluated through the follow-up and side effects and safety were monitored regularly. Compounded drug showed effectiveness and safety. Indeed, during the four-year follow-up, with the dose of adenosine-5′-triphosphate gradually increasing up to 21 mg/kg, the patient showed a substantial benefit in controlling the frequency and duration of hemiplegic episodes and an improvement in neurological deterioration.

## Introduction

Alternating hemiplegia of childhood (AHC, MIM 104290) is a rare neurodevelopmental disorder first described in 1971 (1), with a prevalence of 1/1,000,000 people and an annual incidence of less than 1/100,000 newborns (2, 3).

**Figure 1:**
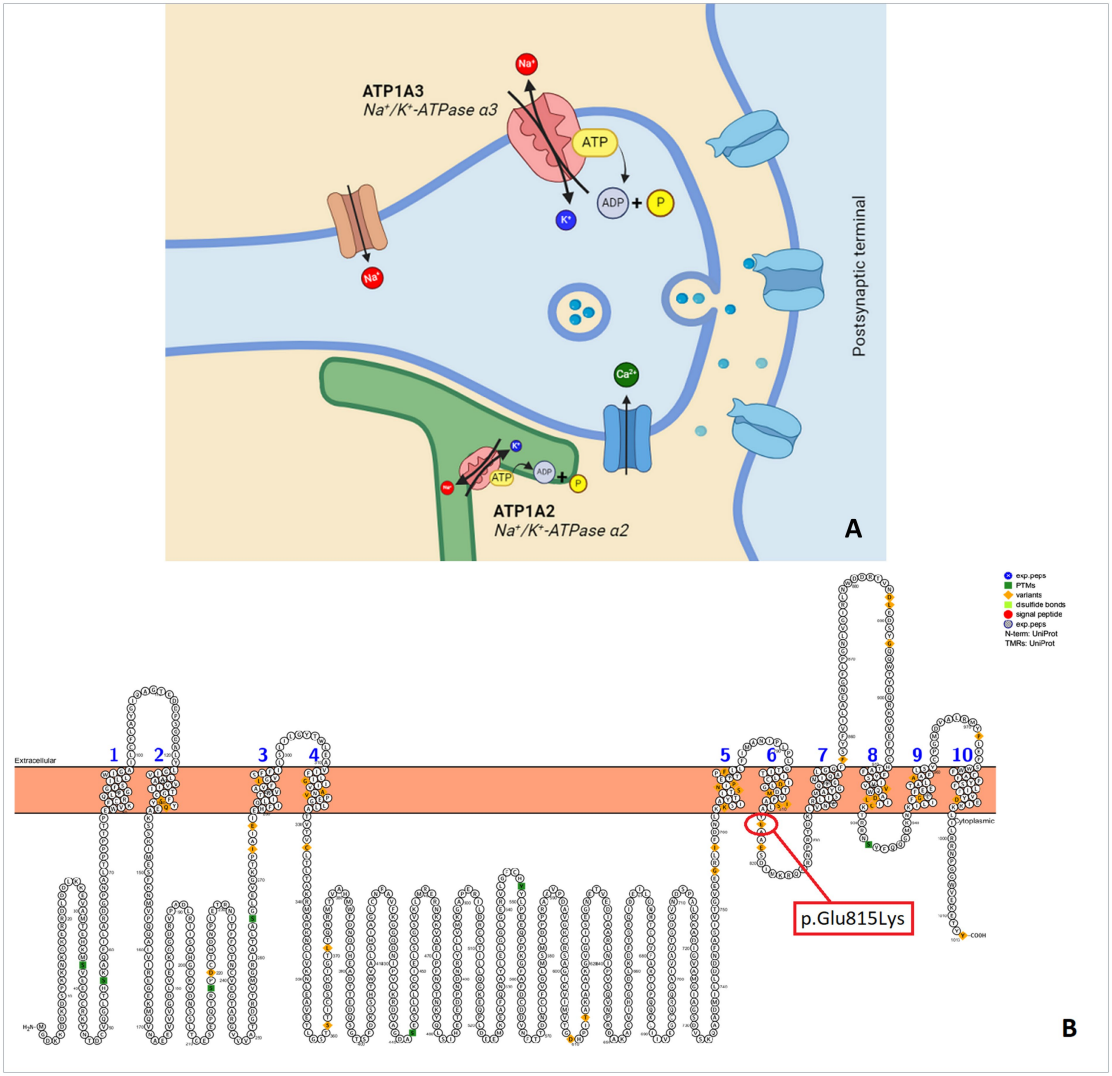
**(A)** Synapse and Na+/K + -ATPase pump. **(B)** Protter illustration of a human ATP1A3 with p.Glu815Lys mutation that occur in our patient (Protter - visualize proteoforms: Omasits et al., Bioinformatics. November 21, 2013).

AHC is due to mutations of ATP1A3 gene. ATP1A3 is the *α* subunit of the transmembrane ion pump Na+/K + -ATPase (Figure 1) that is a protein critical for the proper functioning of neuronal signaling by maintaining physiological sodium (Na+) and potassium (K+) gradients across the plasma membrane of neurons (4). The number of patients and the frequency of onset have increased over the past decade due to the availability of more accessible genetic testing and increased awareness (1).

The onset of AHC usually occurs before 18 months of age and is characterized by heterogeneous symptoms that include paroxysmal and non-paroxysmal disorders. Paroxysmal episodes include: hemiplegic episodes of variable frequency lasting minutes to days involving one or both sides of the body and usually resolving during sleep, epilepsy, dystonic seizures, oculomotor abnormalities, and autonomic phenomena triggered by factors such as environmental stress. Non-paroxysmal signs include cognitive impairment and movement abnormalities such as choreoathetosis, dystonia, or ataxia (1, 5).

The pathophysiological mechanism of AHC is due to the reduced α3 activity that may interfere with reuptake of neurotransmitters such as glutamate, *γ*-aminobutyric acid (GABA) and dopamine (6). Indeed, the activity of Na+/K+ ATPases restoring neuronal membrane potential after depolarization contributes to maintaining neuronal excitability and could support the re-uptake of neurotransmitters.

Adenosine-5′-triphosphate (ATP) is a purine nucleotide which has the function of transferring energy in most of the body’s metabolic processes. Extracellular ATP acting on purinergic P receptors exerts numerous functions such as vasodilation and potential modulation of neuromediators.

Furthermore, a case report hypothesized the potential use of ATP to improve AHC symptoms, demonstrating improvements in balance, fine motor coordination and academic performance (7).

We report a patient with AHC and a *de novo* mutation in ATP1A3 at exon 18 (c.2443 g > A - p.Glu815Lys) who was treated with oral compounded ATP, which decreased the frequency and intensity of hemiplegic episodes.

## Methods

### Case description and data collection

This is a prospective study conducted from February 2020 to November 2023 on a girl born in February 2010. Psychomotor development was assessed with the use of the Bayley III scale (8) and the WISC IV scale for intellectual functioning (9). Paroxysmal Disability Index (PDI - 10), Non-Paroxysmal Disability Index (NPDI - 10) and Clinical Global Impression (CGI - 11) were used for clinical assessment and follow-up. Movement ABC (10) was used to evaluate motor development during treatment, withdrawal, and resumption of treatment. To assess treatment acceptance and effectiveness, the detailed chronic treatment acceptance questionnaire was filled out after the start of ATP therapy.

As there is no ATP-based drug on the market, an ATP compounding has been requested to the Clinical Galenic service of the Hospital Pharmacy of our Institute and a new therapy based on 100 mg ATP capsules has been developed.

The episodes of AHC began to occur from the very first months of life. The genetic analysis has revealed a pathogenic *de novo* mutation at exon 18 c.2443 g > A-p.Glu815Lys, confirming the diagnosis.

The child suffered numerous and repeated episodes of hemi or tetraparesis lasting from 30 min to 3–4 h. Hemiparesis recovered after spontaneous or pharmacologically induced sleep with benzodiazepines (nasal midazolam). To date, no seizures have been reported nor documented. Repeated electroencephalographic examinations during wakefulness and sleep, even during the episodes of hemiparesis were always normal without paroxysmal abnormalities. No respiratory dysfunction or autonomic disturbances have ever been reported or documented. She was also ataxic with polyfocal non epileptic myoclonic jerks which interfered mainly with writing activities and, to a lesser extent, with activities of daily living. Muscle tone decreased overall, but there was no hyposthenia; deep tendon reflexes were normal. There were no ocular motility disorders or dystonic spells. Additionally, parents report that the child tires easily, has poor motor initiative, and has difficulty maintaining attention, especially in school activities. In terms of behavior, parents report that the child is often oppositional with frequent mood swings.

A delay in psychomotor milestones was present: independent ambulation was achieved at age 4 and formulation of sentences of full meaning, at age 8. Psychomotor development was assessed at 14 and 18 months using the Bayley III scale. The worst performance was on the “motor development” subscale, whereas the “cognitive” and “language development subscales” were relatively better, although still below average. (Supplementary Table S1). At age 11 years, the WISC IV scale documented moderate cognitive impairment (IQ 40) with poorer performance on the Fine Motor Index (Supplementary Table S1). Flunarizine and topiramate have been used to treat hemiparetic episodes over the years. Due to ineffectiveness (flunarizine) or adverse effects such as hyperthermia (topiramate), these drugs have been discontinued. The lack of therapeutic alternatives has brought clinicians to start a therapy based on oral compounded ATP administration.

## Results

In February 2020 (age 10 years old, weight 19 kg) a new therapy based on 100 mg ATP capsules (oral once a day after breakfast) compounded by the Clinical Galenic service of the Hospital Pharmacy of our institute has been set up.

**Figure 2:**
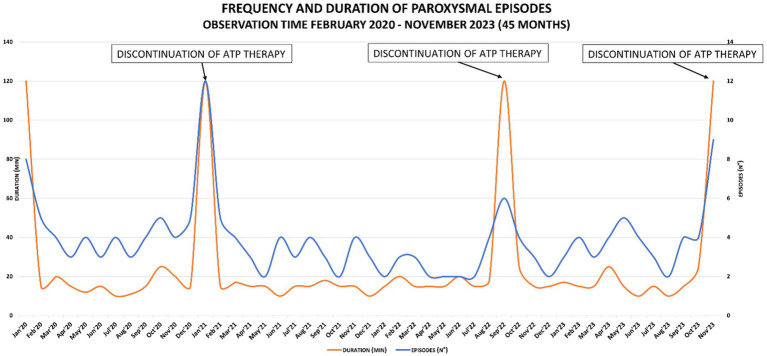
Graph of frequency and duration of paroxysmal episodes: peaks correspond to the discontinuation of ATP therapy.

Meanwhile, rehabilitation treatment (speech and occupational therapy) continued at the territorial services, although the therapy was irregular and discontinuous due to the parents’ organizational difficulties, the child’s lack of cooperation and the difficulties caused by the coronavirus pandemic. In this period family members and rehabilitating staff reported that the child was more toned and active during the subsequent month’s follow-up. No improvement in terms of number of plegic episodes was observed, nevertheless, recoveries were faster. In April, five brief episodes and in May, six more episodes of hyposthenia were reported, all of them mainly focused on the right upper limb. The therapy was confirmed for the following 6 months. In January 2021 physicians decided to suspend the treatment for two weeks to assess clinical progress and confirm that the improvements described were attributable to the ATP treatment and not to the pandemic-related lifestyle change, such as stopping school attendance, increasing sleep hours, and general reduction of environmental stresses. During ATP therapy, a mean of one plegic episode per week was reported. Starting from the first day of ATP therapy discontinuation (day 1), episodes were increased both in frequency and duration: two episodes were reported on day 1, one episode on day 2, no episode on day 3 and again one episode on days 4, 5 and 6. On day 7, the child was admitted to the hospital for clinical evaluations, and 1 to 4 daily hyposthenia episodes lasting from 15 min to 30 min, were reported. The ATP therapy has been therefore resumed. The child’s cognitive-motor skills were then examined in two neuropsychological assessments (Supplementary Table S1), showing improvement in performance. In agreement with parents, it was therefore decided to continue the ATP treatment based on these clinical and neuropsychological findings. During the follow-up of March 2021, a steady increase in the number and duration of paretic episodes (one per week), still localized widespread on the left side, was observed. School performance also improved, and in all social situations, the child was calm, sunny, interactive, and cooperative. Sleep was regular in quality and quantity. Due to the persistence of the episodes and the absence of referred side effects, clinicians decided to increase ATP therapy to a dose of 400 mg/day (200 mg b.i.d. - equal to 21 mg/kg – at breakfast and lunch). The frequency of episodes of alternating hemiplegia, predominantly on the left, lasting approximately 15 min, remained consistent with the new dosing, with one episode per week. The family experienced challenges with administering the drug, which resulted in non-adherence to the treatment for a period of 3 weeks in September 2022. As a consequence, the duration of the attacks increased, with a first attack lasting 3,5 h, a second attack lasting 1 h and a third attack lasting 30 min. At the follow-up of October 2023, the patient apparently refused to take the therapy due to reported abdominal pain. When asked to confirm this, the mother was unsure but was unable to rule it out. The frequency of episodes was stable around 1 episode/week, characterized only by a right or left brachial monoparesis associated with general asthenia, lasting 15–30 min. In agreement with the family members, the therapy was suspended for one month to evaluate the real effectiveness of the ATP therapy. During this time frame, variations in the frequency and duration of episodes of mono/hemiparesis have been recorded, showing an increase up to 9 episodes lasting approximately 2 h. Frequency and duration of paroxysmal episodes over the years and the effects of ATP therapy, are shown in Figure 2.

**Figure 3:**
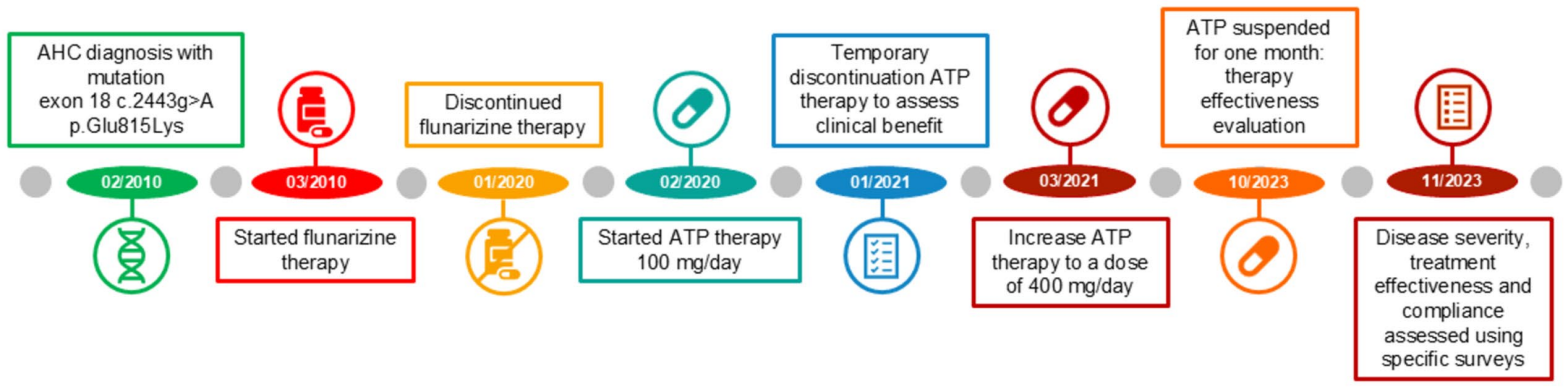
Treatment timeline from February 2010 to November 2023.

The different therapy regimens in chronological order are shown in Figure 3.

The neurological evaluations, the severity of the disease, the effectiveness and acceptance of the treatment were assessed by the neuropsychiatric specialists of our Pediatric Institute in November 2023 using specific surveys.

### Determination of ‘paroxysmal disability index’ (PDI)

**Figure 4:**
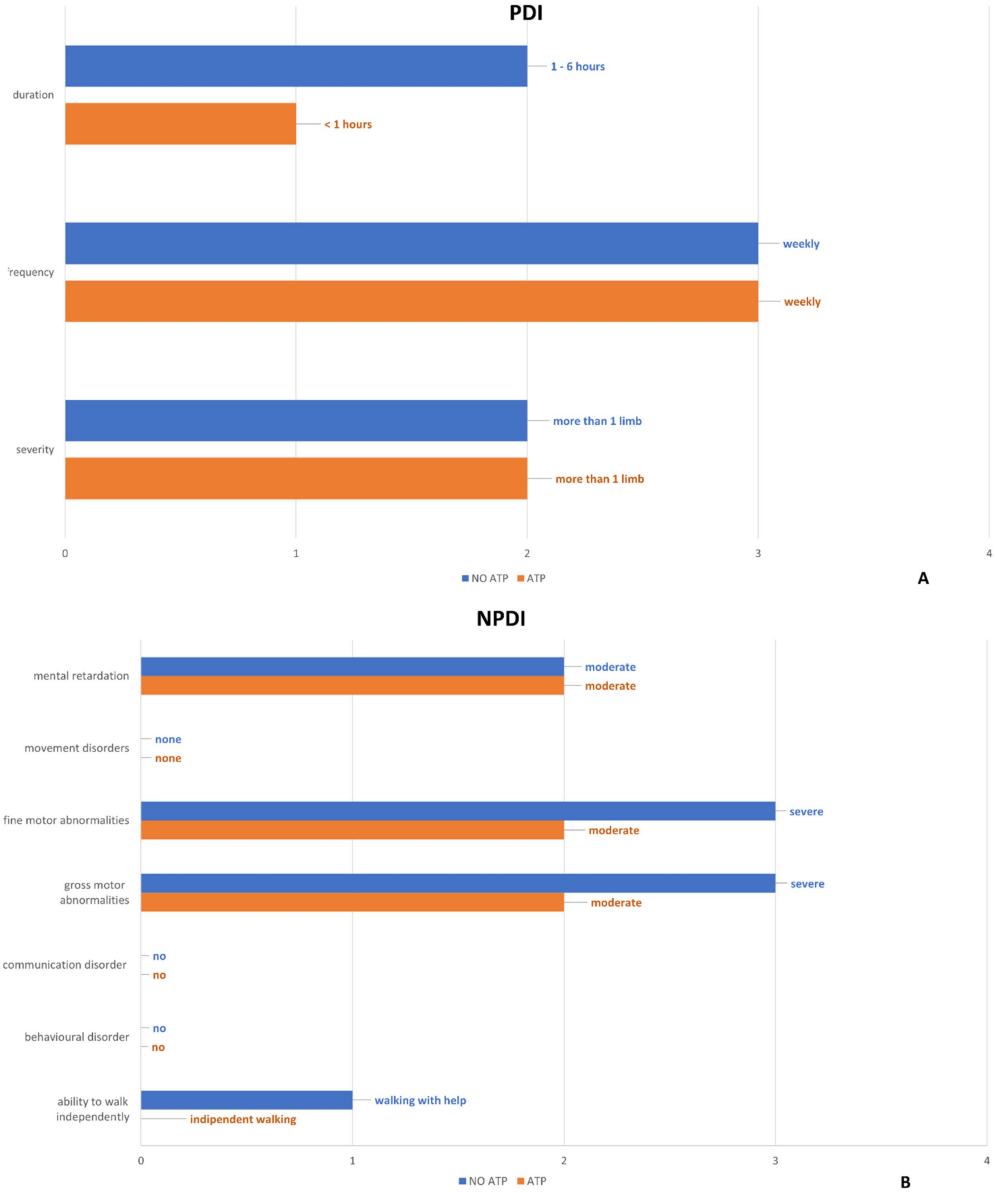
The results of the “Paroxysmal Disability Index” **(A)** and “Non-Paroxysmal Disability Index” **(B)** items are summarized by comparing the values before and after ATP treatment.”

To determine the extent of hemiplegic attacks, PDI assesses their severity frequency and duration (11). The scores assigned by the physician demonstrated a reduction in the patient’s frequency and duration of the episodes after the introduction of the compounded ATP therapy. (Figure 4 and Supplementary Table S2).

### Determination of ‘non-paroxysmal disability index’ (NPDI)

The non-paroxysmal disability index is used to describe the severity of global neurological impairment (11). The non-paroxysmal disability index assessed by the physician has documented an improvement in the items “ability to walk independently,” “gross motor abnormalities” and “fine motor abnormalities” after the introduction of the compounded ATP therapy (Figure 4 and Supplementary Table S3).

### Clinical global impressions (CGI) scale

In November 2023, a CGI survey (12) was submitted to the physician to evaluate the state of the disease and the effect of therapy with the compounded ATP. The result showed a marked improvement with partial remission of symptoms and few side effects (Supplementary Figure S4).

### Evaluation of treatment compliance

We administered a survey to the patient’s caregiver to find out the patient’s experience and opinion regarding ATP therapy. This tool helps to understand the patient’s level of therapy compliance. High adherence to therapy helps maintain high clinical benefits from treatment. The survey highlighted that, despite being conscious that ATP treatment is effective for health improvement, the frequency and duration of the therapy is not easily accepted by the patient. (Supplementary Figure S5).

## Discussion

We reported the case of a patient diagnosed with alternating hemiplegia of childhood caused by ATP1A3 mutations at exon 18 (c.2443 g > A - p.Glu815Lys or E815K) (Figure 1). Pathogenic variants of ATP1A3 are linked to various groups of neurological disorders. To date, several distinct phenotypes have been identified: rapid onset dystonia-parkinsonism (RDP, OMIM 128235), alternating hemiplegia of childhood (AHC, OMIM 614820), cerebellar ataxia-areflexia-cavus foot-optic atrophy-loss syndrome sensorineural hearing (CAPOS, OMIM 601338), developmental and epileptic encephalopathy (DEE, OMIM 619606). Some individuals may exhibit intermediate phenotypes or only some characteristics that do not fit into any of the main phenotypes (13–16).

Alternating hemiplegia may result from mutations in the following genes: ATP1A3, ATP1A2, CACNA1A, SLC1A3 and SLC2A1; AHC is a distinctive syndrome, and very few if any children with these mutations meet full diagnostic criteria for AHC. However, mutations in the neuronal Na+/K+ ATPase gene ATP1A3 have been found in 75–85% of cases (1, 3, 5, 17).

Individuals with AHC and the pathogenic p.Glu815Lys variant in the neuronal Na+/K+ ATPase may have an earlier onset of symptoms and greater motor and cognitive impairment, and more often experience status epilepticus and respiratory paralysis (13). Paroxysmal disorders that patients may present include epilepsy, dystonic seizures, oculomotor abnormalities, and autonomic phenomena; non-paroxysmal signs are cognitive impairment and movement disorders, such as choreoathetosis, dystonia, or ataxia. The more severe intellectual and motor disability phenotype was associated with p.Glu815Lys. The phenotypic expression of p.Asp801Asn was milder, while p.Gly947Arg was associated with the most favorable prognosis (17). In some cases, patients with this mutation presented rapid motor decline and language loss and a sudden worsening of dystonia (18, 19).

ATP1A3 mutations can be varied: 34 distinct ATP1A3 mutations were found, grouped into five distinct regions overall. The ATP1A3 mutations that occur most frequently are p.Glu815Lys (16%), p.Gly947Arg (11%), and p.Asp801Asn (43%). ATP1A3 α3 subunit (together with ATP1A1, ATP1A2 subunits) are mainly expressed in interneurons, pyramidal (17), basal ganglia, hippocampus, and cerebellum cells (https://omim.org/entry/182350) suggesting that they play important roles in the brain. Furthermore, Na+/K + -ATPase (NKA) α3 showed co-localization with markers of different populations of GABAergic neurons in rodent and human brains. The NKA complex is responsible for exchanging three cytoplasmic sodium ions for two extracellular potassium ions, thereby establishing electrochemical gradients essential for critical cellular functions and membrane potential (20). It plays an important role in the rapid restoration of basal intracellular Na + concentrations after high neuronal activity (21).

Even though the E815K mutation is at the cytoplasmic domain, however E815K resides more in the transmembrane domain than in the cytoplasmic domain (22). These data suggest that ATP1A3 mutations causing AHC modulate the Na+/K + -ATPase pump’s activity, impairing ion transport including loss of proton transport availability particularly in the case of E815K mutations (20). None of the mutations affected the mRNA expression of ATP1A3. So AHC-causing mutations revealed consistent reductions in ATPase activity without effects on protein expression. *In vitro* studies of mutations causing ACH show reduced ATP 1A3 activity ranging from 54 to 90 percent (19). The activity of Na+/K + -ATPases restoring neuronal membrane potential after depolarization contributes to maintaining neuronal excitability and could support the re-uptake of neurotransmitters and the reduced α3 activity may interfere with reuptake of neurotransmitters such as dopamine, glutamate, and *γ*-aminobutyric acid (GABA) supporting GABA dysfunction (6). Finally, in a knock-in mouse model of alternating hemiplegia, it was hypothesized that mutations in the ATP1A3 subunit may cause an abnormal neuronal predisposition to spreading depression, which could explain the motor deficit typical of this form (23).

The clinical features of our patient compared with those reported in the literature in subjects with the same mutation show some similarities but also some differences. In literature typical symptoms of this mutation are intellectual disability, walking problems, ataxia, dystonia, hemiplegia/double hemiplegia attacks, tonic attacks, and epilepsy (17, 22, 24, 25). Other reported symptoms include ocular motility disorders respiratory dysfunction and autonomic disturbances. Our patient presents only with intellectual disability, walking problems, ataxia, and hemiplegia. Motor symptoms improved after the start of ATP treatment (especially at the 21 mg/kg dose) and worsened when the drug was discontinued (Figure 4). Indeed, walking improved, gross and fine motor skills improved, and episodes of hemiparesis became less intense (Figure 4), with a reported improvement in quality of life. Interestingly, parents reported that general mood improved during treatment, and the child became more active and resistant to fatigue.

Regarding the dosage, therapy was started at a low dose. This was done to check for side effects. After evaluating the efficacy of the drug and the absence of side effects, the dosage was increased to 21 mg/kg.

### Therapy

Disease-modifying therapy of alternating hemiplegia of childhood does not exist, and several agents such as flunarizine, topiramate, ketogenic diet, triheptanoin, steroid, amantadine, memantine, aripiprazole, oral ATP, coenzyme Q, acetazolamide, dextromethorphan, and vagus nerve stimulator have been tried with various rates of success by aborting attacks or reducing the frequency or severity of paroxysmal spells (2). Flunarizine is the most used therapy in the prevention of hemiplegic episodes as it can reduce their frequency, severity and duration. It is a non-selective calcium channel blocker, and rather than acting directly on the pump, it may inhibit intracellular calcium accumulation and provoke cerebral vasodilation. Its effectiveness is variable ranging from 50 to 70% in different case studies (2, 26). In our patient, flunarizine was ineffective in modifying the symptomatology, and benzodiazepines to induce sleep had resolved the motor symptoms. Topiramate was ineffective with significant side effects (hyperthermia).

A study reported the use of oral ATP in a six-year-old child with AHC (*de novo* ATP1A3 mutation) (7). Dose-dependent reduction of frequency, duration, and severity of the hemiplegic episodes were noted during one-year follow-up when authors increased the oral ATP dose from 2 to 25 mg/kg/day. The authors hypothesized that ATP supplementation might help the dysfunctional ATPase and ATP might increase the mechanical strength of the hemiplegic limb by increasing blood flow secondary to its vasodilatory property (2).

Extracellular ATP, directly or as ADP, activates P2 purinergic receptors, which are divided into two subclasses, PX and PY. The molecules that regulate purinergic signaling, including receptors, channels, enzymes and transporters, are expressed by both neurons and glia. Some of these molecules are expressed in specific cell types (e.g., P2Y12 receptor in microglia). Accumulating evidence indicates that purinergic signaling plays a key role in neuron–glial communication in the brain (27). Furthermore, previous studies have shown that extracellular ATP can modulate GABAAR receptor function by activating P2 receptors (28), although more recent evidence suggests a direct effect of extracellular ATP on GABAergic receptors by enhancing the inhibitory effect of GABA (29). Regarding vasodilation, purinergic P2Y receptors activated by extracellular ATP mediate endothelium-dependent vasodilation mainly through the release of nitric oxide and partly through prostaglandins (30). ATP therapy improved some of our patient’s symptoms reducing frequency and intensity of hemiparesis, better motor performances as confirmed by CGI scales. Furthermore, we did not document worsening, as described in some N815K mutation cases (6, 31, 32). We can speculate that extracellular ATP exerted its function by mediating both vasodilating and GABAergic effects. In the latter case ATP appears to behave, at least in part, like benzodiazepines by increasing GABA-mediated inhibition (33) but without their sedative effect. In our patient, pharmacologically induced sleep with benzodiazepines resolved the acute symptoms and the GABAergic effect of ATP could explain its efficacy. We cannot rule out a placebo effect, although the objective observation of the parents and rehabilitating staff regarding the improvement of the motor symptoms and its worsening on discontinuation of the treatment may indicate a therapeutic effect. Children with genetic cognitive disabilities, especially those with lower IQ, as in the case of our patient, are sensitive to the placebo effect, but in close dependence on the acceptance of the family context (34). We speak of “placebo by proxy” when the attitude of the context is positive regarding the treatment, but also of “nocebo by proxy” when the attitude of the context is negative, which in turn can negatively influence the patient’s response to the treatment (35). In this case, the mother’s responses to the survey (Supplementary Figure S4) indicate that, given a positive attitude regarding efficacy, there are more negative critical aspects related to accepting the treatment and continuing with it, as evidenced by nonadherence to treatment in September 2022. We could therefore think of a “nocebo by proxy” type effect. This negative attitude could have negatively affected the child’s symptomatology upon resumption of therapy, which did not occur, suggesting a possible treatment effect rather than a placebo effect.

This report has limitations because it is a single case report and not a randomized, double-blind, controlled trial (RCT). However, as this is a rare disease, RCTs may not always be feasible. Critical issues include the absorption of ATP at the gastrointestinal level, its distribution and bioavailability, including in the central nervous system, and not least the variability of symptomatology, which is well known in this syndrome. Regarding ATP absorption and systemic bioavailability, reviews show that oral ATP can improve muscular strength and general exercise performance (36, 37). As far as the central nervous system is considered, a recent study showed that oral ATP supplementation in athletes can improve some neuropsychological variables, such as visuomotor reaction time and cognitive function (38).

As with our patient, no significant adverse or related side effects have been reported in the literature regarding the safety of oral ATP supplementation (36, 39).

## Conclusion

This case demonstrated that oral compounded ATP therapy could be a useful alternative for treating alternating hemiplegia of childhood caused by ATP1A3 mutations after failure of conventional medication. Furthermore, the stabilization of symptoms and lack of deterioration may be indicative of a potential ‘disease-modifying therapy’ effect of ATP.

Even with its limitations this is a clinical article that attempts to combine the clinical findings with some pharmacological and biochemical working hypotheses that could or should be the subject of further studies in larger populations, both with the same mutation and with other mutations.

However, this Case Report also highlights the valuable role of Hospital Pharmacy, with its expertise, in solving drug availability problems, particularly in cases where drugs are not commercially available, thus enabling treatment of rare diseases.

## Data Availability

The raw data supporting the conclusions of this article will be made available by the authors, without undue reservation.
