# Supplementary material for: Oral ATP treatment in alternating hemiplegia of childhood: a case report and review
Source: Front Med (Lausanne). 2025 Jan 7;11:1433217. doi: 10.3389/fmed.2024.1433217 (PMC11747781; doi:10.3389/fmed.2024.1433217)

Supplementary Material

**Oral ATP treatment in alternating hemiplegia of childhood. (A case report and review)**

**Frontiers in Medicine**

**Marco Carrozzi, Maria Elisa Morelli, Mario Cirino*, Alessandra Maestro, Gilda Paternuosto, Giulia Benericetti, Giada Bennati, Anna Flamigni, Federico Pigato, Natalia Maximova, Egidio Barbi, Davide Zanon**

*** Correspondence:** Mario Cirino [mario.cirino@burlo.trieste.it](mailto:mario.cirino@burlo.trieste.it)

# Supplementary Tables and Figures

**Supplementary Table S1.** Psychomotor development was assessed with the use of the Bayley III scale and the WISC IV scale for intellectual functioning. The ABC of Movement was used to assess motor development during treatment, treatment discontinuation and treatment resumption.

| **Age** | **Test/Scaled** | **Drug** | **Total Development Score** | **Cognitive Development Subscore** | **Language Development Subscore** | **Motor Development Subscore** |
| --- | --- | --- | --- | --- | --- | --- |
| 14 months | Bayley III scale | flunarizine  5 mg | / | 80 | 83 | 61 |
| 18 months | Bayley III scale | flunarizine  5 mg | / | 80 | 83 | 58 |
| 11 years | WISC IV | none | 40 (ID) | 52  (perceptual reasoning index) | 52  (verbal comprehension index) | 50  (fine motor index) |
|  | | | | | | |
| **Movement ABC-2** | | | | | | |
|  |  |  | **Manual Dexterity Subscore** | **Aiming & Catching Subscore** | **Balance Subscore** | **comment** |
| 10 years |  | ATP  300 mg/day | 2  (standard score)*  < 0.5 percentile | 3  (standard score)*  1 (percentile) | n.a. | inferior normal |
| 11 years |  | none | 2  (standard score)  0.5 (percentile) | 2  (standard score)  0.5 (percentile) | n.a. | worse |
| 11 years |  | ATP  400 mg/day | 2  (standard score)  0.5 (percentile) | 5  (standard score)  5 (percentile) | n.a. | inferior normal but improved |
| * standard score: 10 ± 3 ds  n.a.: not assessable | | | | | | |

*[Bayley N. In: Bayley Scales of Infant and Toddler Development. 3rd ed. Ferri R., Orsini A., Stoppa E., editors. Giunti Psychometrics; Firenze, Italy (2006) - Orsini A., Pezzuti L., Picone L. WISC-IV: Contributo Alla Taratura Italiana. Giunti O.S.; Florence, Italy: 2012. [WISC-IV Italian Edition] - Biancotto et al., 2017; M. Biancotto, M. Guicciardi, G.M. Pelamatti, T. Santamaria, S. Zoia Movement Assessment Battery for Children-second edition. standardizzazione Italiana Giunti O.S. Psychometrics, Firenze (2017)]*

**Supplementary Table S2.** The detailed questionnaire filled out by the physician for the evaluation of Paroxysmal Disability Index (PDI). The evaluation was performed before and after the start of ATP therapy. *(Adapted from Eleni Panagiotakaki, et al. Brain, Volume 133, Issue 12, December 2010, Pages 3598–3610)*

| **Paroxysmal disability index (PDI)** | | **Before ATP treatment** |
| --- | --- | --- |
| Severity  (number of extremities involved) | one limb |  |
|  | more than one limb | 2 points |
|  | both sides or 4 limbs |  |
| Frequency | <1 attack/year |  |
|  | monthly attacks |  |
|  | weekly | 3 points |
|  | daily |  |
| Duration | <1 hours |  |
|  | 1–6 hours | 2 points |
|  | 6–12 hours |  |
|  | 12–24 hours |  |
|  | >24 hours |  |

| **Paroxysmal disability index (PDI)** | | **After ATP treatment** |
| --- | --- | --- |
| Severity  (number of extremities involved) | one limb |  |
|  | more than one limb | 2 points |
|  | both sides or 4 limbs |  |
| Frequency | <1 attack/year |  |
|  | monthly attacks |  |
|  | weekly | 3 points |
|  | daily |  |
| Duration | <1 hours | 1 point |
|  | 1–6 hours |  |
|  | 6–12 hours |  |
|  | 12–24 hours |  |
|  | >24 hours |  |

# Supplementary Table S3. The detailed questionnaire filled out by the physician for the evaluation of Non-Paroxysmal Disability Index (NPDI). The evaluation was performed before and after the start of ATP therapy. *(Adapted from Eleni Panagiotakaki, et al. Brain, Volume 133, Issue 12, December 2010, Pages 3598–3610)*

| **Non-paroxysmal disability index (NPDI)** | | **Before ATP treatment** | **After ATP treatment** |
| --- | --- | --- | --- |
| ability to walk independently | independent walking |  | 0 points |
|  | walking with help | 1 point |  |
|  | not possible |  |  |
| behavioural disorder | no | 0 points | 0 points |
|  | yes |  |  |
| communication disorder | no | 0 points | 0 points |
|  | yes |  |  |
| gross motor abnormalities | none |  |  |
|  | mild |  |  |
|  | moderate |  | 2 points |
|  | severe | 3 points |  |
| fine motor abnormalities | none |  |  |
|  | mild |  |  |
|  | moderate |  | 2 points |
|  | severe | 3 points |  |
| movement disorders  (chorea, dystonia, myoclonus, tremor and complex movement disorders) | none | 0 points | 0 points |
|  | mild |  |  |
|  | moderate |  |  |
|  | severe |  |  |
| mental retardation | none |  |  |
|  | mild |  |  |
|  | moderate | 2 points | 2 points |
|  | severe |  |  |

**Supplementary** **Figure S4.** The detailed questionnaire filled out by the physician for the evaluation of Clinical Global Impression (CGI). The evaluation was performed after the start of ATP therapy**.** *(Adapted from Guy W. “Clinical Global Impressions”. In: Guy W Ph.D. editor. ECDEU Assessment Manual for Psychopharmacology. Rockville, MD, U.S. Department of Health, Education, and Welfare (revised 1976). p. 217-222.*


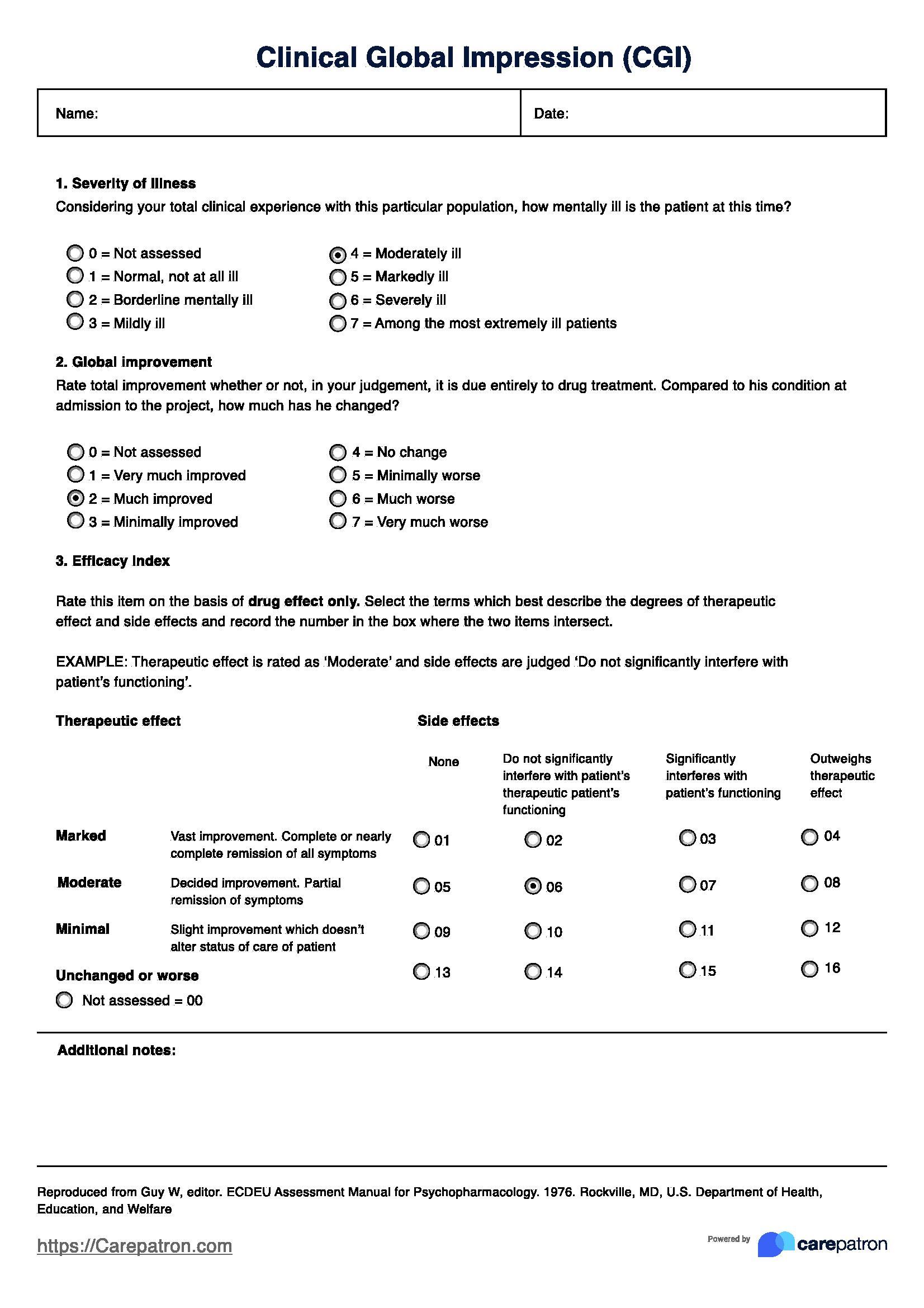


**Supplementary Figure S5.** The detailed chronic treatment acceptance questionnaire filled by the patient for compliance assessment. The evaluation was performed after the start of ATP therapy**.**
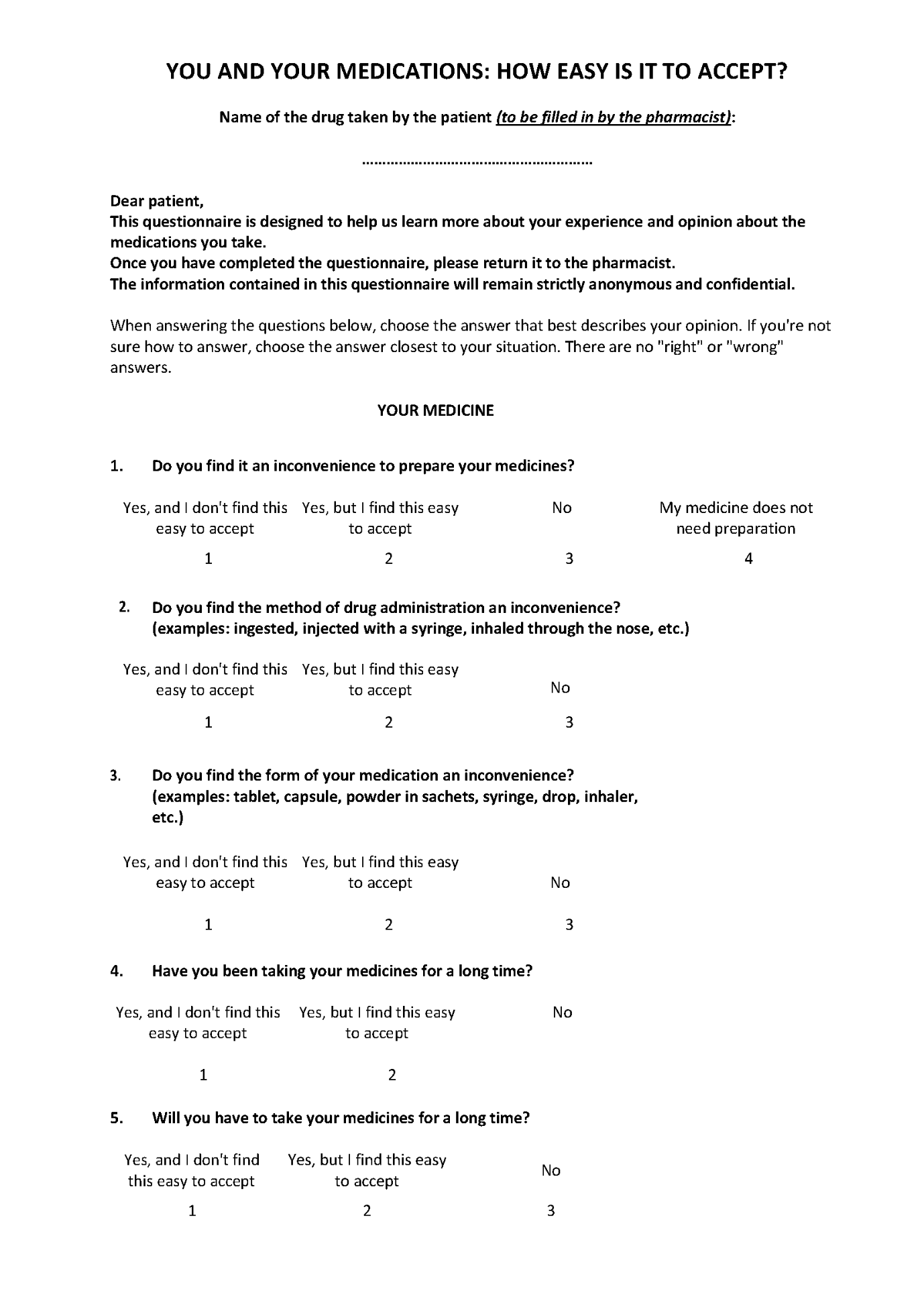


3


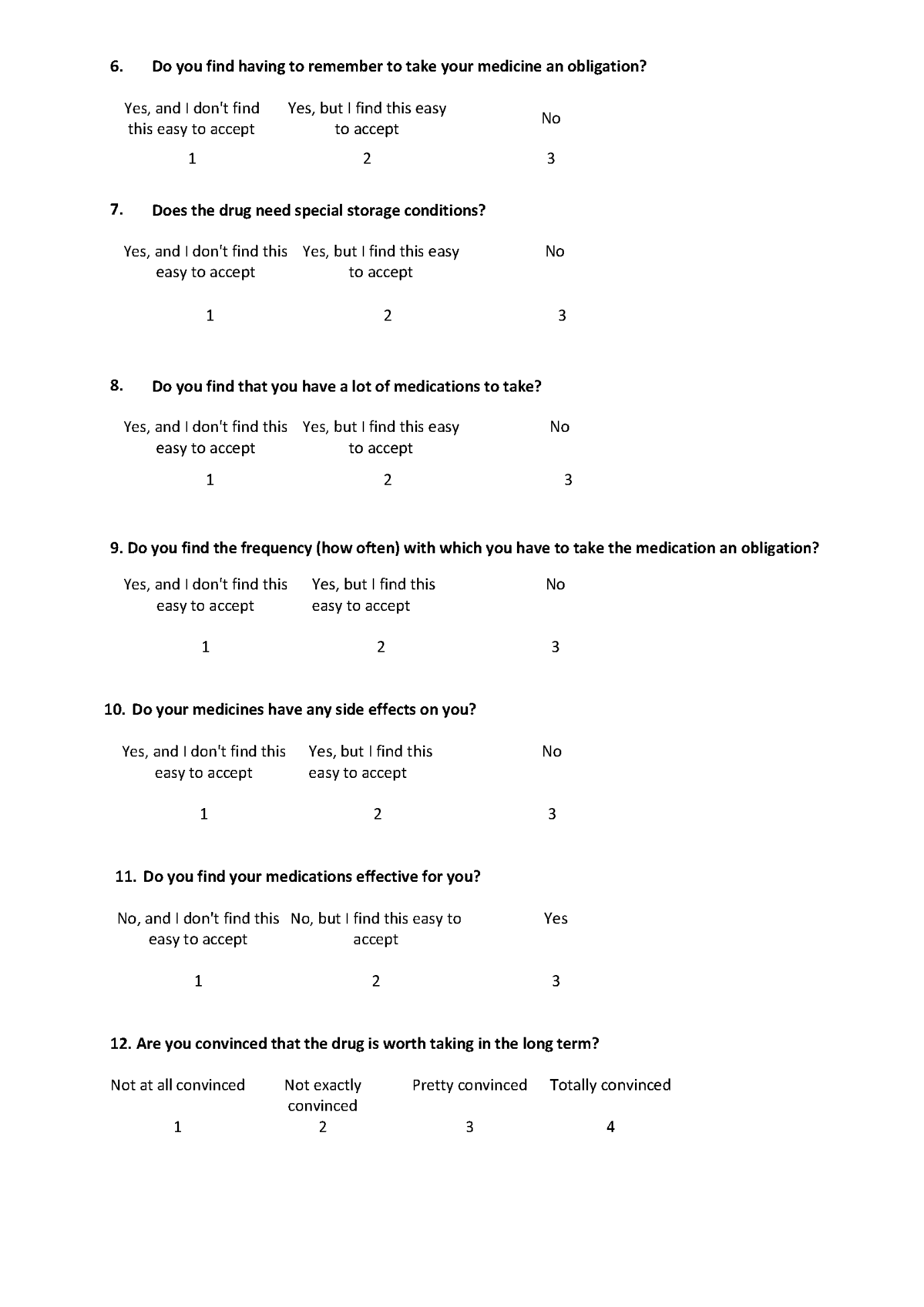

Supplement: Supplementary file 1 [file Data_Sheet_1.docx]
